# Characterization of Basil Volatile Fraction and Study of Its Agronomic Variation by ASCA

**DOI:** 10.3390/molecules26133842

**Published:** 2021-06-24

**Authors:** Alessandro D’Alessandro, Daniele Ballestrieri, Lorenzo Strani, Marina Cocchi, Caterina Durante

**Affiliations:** 1 Barilla G. e R. Fratelli, Via Mantova 166, 43122 Parma, Italy; alessandro.dalessandro@barilla.com (A.D.); daniele.ballestrieri@barilla.com (D.B.); 2 Department of Chemical and Geological Sciences, University of Modena and Reggio Emilia, Via Campi 103, 41125 Modena, Italy; lostrani@unimore.it (L.S.); cdurante@unimore.it (C.D.)

**Keywords:** basil, aroma, fast GC, GC/O, electronic nose, PCA, ASCA, cut, variety

## Abstract

Basil is a plant known worldwide for its culinary and health attributes. It counts more than a hundred and fifty species and many more chemo-types due to its easy cross-breeds. Each species and each chemo-type have a typical aroma pattern and selecting the proper one is crucial for the food industry. Twelve basil varieties have been studied over three years (2018–2020), as have four different cuts. To characterize the aroma profile, nine typical basil flavour molecules have been selected using a gas chromatography–mass spectrometry coupled with an olfactometer (GC–MS/O). The concentrations of the nine selected molecules were measured by an ultra-fast CG e-nose and Principal Component Analysis (PCA) was applied to detect possible differences among the samples. The PCA results highlighted differences between harvesting years, mainly for 2018, whereas no observable clusters were found concerning varieties and cuts, probably due to the combined effects of the investigated factors. For this reason, the ANOVA Simultaneous Component Analysis (ASCA) methodology was applied on a balanced a posteriori designed dataset. All the considered factors and interactions were statistically significant (*p* < 0.05) in explaining differences between the basil aroma profiles, with more relevant effects of variety and year.

## 1. Introduction

Basil (*Ocimum basilicum* L.) is an annual plant of the *Lamiaceae* family, known worldwide as a culinary and healthy herb [1]. Basil’s essential oils have been used in many fields for medicinal treatments, perfumery and cooking spices. Originating from India, Africa and Asia, its cultivation is now spread worldwide [2].

It is estimated that basil counts from fifty to one hundred fifty species, of which the most commonly used in the culinary field is sweet basil [3,4]. It is present in many different chemo-types due to its characteristic to easily cross-breeds [4,5]. For that reason, it can sometimes be challenging to determine the species or the variety of a basil plant. Its characteristics in terms of morphology, agronomy performances and aroma pattern are normally determined [6,7,8]. These characteristics are influenced not only by the chemotype/species/variety, but also by agronomic practices, climatic conditions and age of the plant [1,3].

The basil aroma is composed of a large number of molecules, mainly terpenoids, alcohols, aldehydes, ketones and esters [3,9]. Totally, there are more than one hundred molecules, of which the most representatives in sweet basil are considered linalool, estragole, eugenol and eucalyptol (1,8-cineole) [7,10]. The content of these molecules could give a preliminary evaluation of different basil flavour profiles, while a more accurate evaluation of the final aroma will also consider the concentrations of other minor components, mainly the molecules that have a low odour threshold [11,12]. The odour threshold is defined as the lowest concentration of a molecule that could be perceived by olfaction. Thus, in the evaluation of the flavour patterns, it is necessary to consider not only the concentration of a given molecule but also its capacity to be perceived.

Basil is one of the main components of the “Pesto Genovese” sauce, a typical and well appreciated Italian green sauce. The basil aroma pattern is crucial for the organoleptic features of pesto sauce and consequently its analytical characterization is relevant [13] in terms of selecting the preferred profile or to search for new patterns.

There are many different methods to identify and quantify volatile organic compounds (VOCs) based on gas chromatography (GC) and mass spectrometry (MS), either coupled or not, and using different systems of sampling. Among coupled GC–MS methods, different systems are available to collect, trap and concentrate the VOCs, such as headspace solid phase microextraction gas chromatography–mass spectrometry (HS-SPME-GC–MS) [6], headspace sorptive extraction gas chromatography–mass spectrometry (HSSE GC–MS) [13], dynamic headspace-thermal desorption–gas chromatography/mass spectrometry (DH-TDU-GC–MS) [14]. Direct-injection mass spectrometry (DIMS) [15], without a separation step, is also very diffuse in food analysis [16,17,18]. In particular, the development of an ambient ionization mass spectrometer (AMS) [19,20,21,22,23] is very important, especially coupled with the development of miniature and portable mass spectrometers [21,22,23] and innovative introduction systems, such as membrane inlet mass spectrometers (MIMS) [21,24]. AMS, while opening up very interesting perspectives for in situ food analysis and control, has still to become an established reference for quantitative analysis, especially for solid samples [19].

The basil aroma pattern, to the best of our knowledge, has been characterized only by GC based techniques, for instance, headspace solid phase microextraction gas chromatography–mass spectrometry (HS-SPME-GC–MS) [6], headspace sorptive extraction gas chromatography–mass spectrometry (HSSE GC–MS) [13], as well as gas chromatography as such (GC and GC–MS) [10], indirectly measuring the total phenolic compounds [25] or using flow-injection mass spectrometry [18].

As basil is a very delicate plant, which is difficult to store after cutting [26,27], it would be extremely useful to have a fast analytical method, being at the same time suitable to discriminate the different varieties and furnishing information on the compositional profile of the aroma fraction.

To this aim, in this paper, we tested an electronic nose system based on ultrafast gas chromatography (fast-GC) since it can provide a non-invasive, rapid, sensitive and relatively low-cost system. Moreover, it allows direct comparison with sensory evaluation that is usually carried out by gas chromatography–olfactometry (GC/O) [28] analysis. In particular, the Heracles II e-nose device [29] was tested, which has been previously applied to characterize the volatile fraction of different food commodities [30,31,32,33], while there is, to the best of authors’ knowledge, no study concerning basil or other spices. The aroma profile gathered by fast-GC was matched with sensory evaluation from GC/O, and the detected molecules, mainly perceived in the basil flavour pattern and persistent in GC/O, were quantified.

The developed methodology was applied to evaluate several basil varieties, grown on open fields in different years considering more cuts, to obtain a preliminary overview by multivariate exploratory data analysis of the aroma variation due to both varieties and period of harvesting. A deepest insight and a better understanding of these effects can be gathered by ANOVA–Simultaneous Component Analysis (ASCA) [34], which generalizes classical analysis of variance (ANOVA) to multivariate data, overcoming the main limitations (number of samples higher than number of variables, breakdown in case of variables collinearity) and multinormal distribution assumption of multivariate ANOVA (MANOVA). First, a classic ANOVA was carried out to split the data matrix into the effect matrices for each experimental factor and their interactions. Then, simultaneous component analysis was carried out on the effect matrices to identify and visualize the contribution of the measured variables to each of the effects that introduced systematic variation [35]. One of the main advantages of ASCA is the interpretation of the factor levels in terms of the measured variables through loadings inspection. ASCA has been successfully applied in metabolomics [34,35], as well as in food analysis [36,37,38].

ASCA requires data coming from an experimental design, and thus we applied it to a balanced reduced set of varieties, in order to investigate the effects of cutting period, basil variety and harvesting year on the basil aroma pattern.

## 2. Materials and Methods

### 2.1. Basil Plants

The plants of basil (*Ocimum basilicum*) of twelve different commercial varieties of “genovese” type were supplied, for all the samples, by local producers (Parma Vivai). The varieties name is indicated with a code for confidentiality reasons. Only the “Italiano Classico” has been indicated because it is largely commercially used. All plants have been grown in open fields following standard agricultural practices. Each basil variety was collected at different plant ages: in most cases two cuts were collected and sometimes up to four cuts were taken (Table 1). Plants were cut leaving about 5–6 cm from soils, allowing the plant to regrow for the next cut. The first cut was carried out when the plants were aged 40 days, while the subsequent cuts were carried out at time intervals of about 20 days each. Finally, in order to have a preliminary idea on the variation of the investigated aroma fraction as a function of the harvest, different basil varieties were collected for three years (2018–2020). In Table 1, the number of samples per year, variety and cut are reported.

### 2.2. Sample Preparation

Basil plants were collected early in the morning, typically from 4 to 8 a.m., and were immediately sent to the lab for the evaluations. Plants were analysed within 6–8 h from the cut. About 30 g was exactly weighted at 0.1 g of the whole basil plant, including leaves and stems, and was hashed in a blender (Oster, Sunbeam Products Inc., Boca Raton, FL, USA) for 30 s in 300 mL of extraction solution at room temperature. The extraction solution was prepared with NaCl at a concentration of 100 g L^−1^, to increase the volatiles release in the headspace (next step of the analysis), and 6 mg kg^−1^ of ethyl iso-butyrate to serve as internal standard for the CG analysis. After 30 s of resting time, 20 μL of the solution was collected and transferred in 20 mL amber vials that were immediately sealed and sent for analysis. Each extract was sampled at least three times in different vials. All reagents, standard and solvents were analytical grade (Sigma Aldrich, Inc., Saint Louis, MO, USA).

### 2.3. Heracles e-Nose Analysis

The analysis of the volatile molecules in the sample headspace was carried out using a Heracles II (Alpha MOS, Tuluse, France) ultra-fast chromatography electronic nose [18]. The instrument consists of a double-columns ultra-fast-chromatography system, with FID detectors, interfaced with a PAL-RSI automatic headspace autosampler, after injection a Tenax TA polymer trap is employed. The two columns were mounted in parallel in the oven; they had different polarities, namely, an MXT-5 (non-polar) and MXT-1701 (slightly polar) were employed, both 10 m in length, with internal diameters of 0.18 mm and phase thicknesses of 0.40 μm. A temperature ramp was employed, starting from 50 °C for 2 s, then going to 80 °C at 1 °C·s^−1^ and finally reaching 250 °C at 3 °C·s^−1^. The total fast GC analysis time was 110 s. The carrier gas was hydrogen.

The different replicates of each extracted sample were loaded in the instrument autosampler and incubated for 20 min at 40 °C before injection with 500 rpm agitation (5 s on, 2 s off). Then, 1 mL of air headspace was injected with a syringe temperature of 50 °C. Trap loading conditions were 18 s at 40 °C, then flashed to 250 °C for the release in the two columns at split ratio 1:1.

The AlphaSoft v 16.0 software was used to process the data. Volatile compounds were identified on the basis of Kovats’ relative retention indices (KI) and can be linked to specific molecules that are collected in the AroChemBase v 7.0 database (Alpha MOS., Tuluse, France). In this way, eighteen compounds were tentatively identified as further discussed in Section 3.

### 2.4. Gas Chromatography–Mass Spectrometry Olfactometry Analysis (GC–MS/O)

To select the key molecules perceived in basil aroma, a preliminary analysis on the Italiano Classic variety was conducted by gas chromatography–mass spectrometry coupled with a Gerstel ODP3 sniffing port olfactometer (GC–MS/O). Among the about one hundred and fifty molecules observed in GC–MS (data not shown), only thirty-two were perceived by GC/O sniffing trained panellists in terms of odour, and just nine of these had shown a persistent odour after three dilution steps. Matching these molecules with the eighteen molecules observed in the Heracles chromatograms, nine key marker molecules were selected as the most representative of the basil flavour pattern, as reported in Table 2.

### 2.5. Quantification of Key Molecules

**Figure 1:**
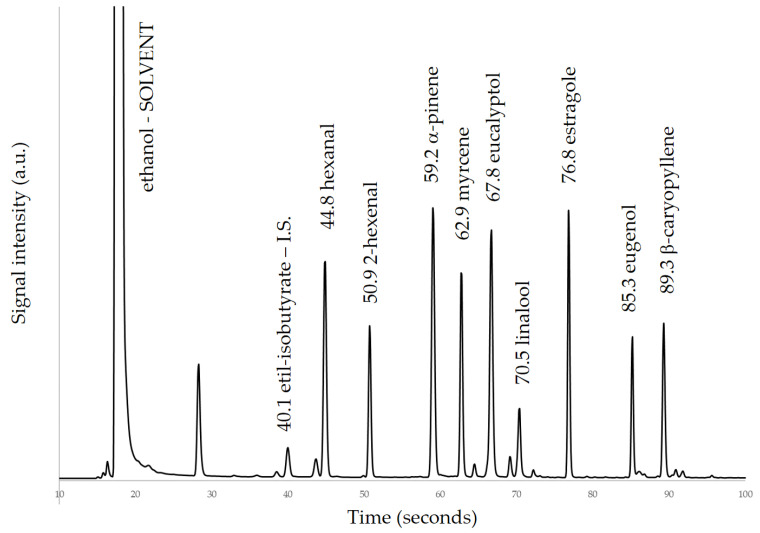
Chromatogram of multistandards solution. The peaks of the nine molecules with their retention times are shown, together with the peak of internal standard (IS) and solvent. Peak just before 30 s and other minor peaks are solvent impurities.

For each of the key nine molecules of interest, a calibration curve was obtained (Table 3) by preparing standard solutions at six concentration levels, using ethyl iso-butyrate as internal standard. Two mother solutions were prepared. First, a solution of ethyl iso-butyrate (internal standard) was prepared by diluting about 100 mg in ethanol, exactly weighed, in a 100 mL volumetric flask to obtain a final concentration of about 1000 mg kg^−1^. The second solution of multistandards was prepared by diluting, in 100 mL of ethanol, quantities of each standard exactly weighed from 60 to 150 mg, depending on the respective standard volatility, to obtain final concentrations ranging from 600 to 1200 mg kg^−1^. The six calibration solutions at different level concentrations were prepared by diluting with ethanol to 5 mL final volume, 0.5 mL of the IS solution and, respectively 0.25, 0.5, 1.0, 1.5, 2.0 and 3.0 mL of the multistandards solution. From each calibration solution, 1 µL was collected and loaded into the 20 mL vials for the analysis. The calibration curve was obtained normalizing the area of each analyte with respect to the internal standard area and quantity. A representative chromatogram for one of the multistandards solutions used for calibration is shown in Figure 1.

As far as the nine investigated compounds are concerned, the calibration curves were linear over the examined concentration range. In Table 3, the coefficient of determination, the slope and the limit of detection (LOD) for each calibration curve are reported.

At the start of a new analytical batch, three empty vials were injected as blanks to clean the system and one empty vial was run between each group of replicates of the samples to assure the system was clean and prevent cross-contaminations between different samples.

The concentration of each molecule was calculated with respect to the exact weight of the plant basil extracted. As a result, a dataset of the concentration in µg kg^−1^ of all the nine marker molecules of the basil samples was obtained.

In order to evaluate the short-term (intra-day) and long-term (inter-day) reproducibility, nine replicates of the same basil sample were prepared from scratch and analysed in the same day at different times, and in three different days, respectively. The relative standard deviation (RSD) was then computed for both reproducibility conditions. In particular, intra-day RSD ranged between 4 and 9%, while inter-day RSD ranged between 8 and 10%, showing good reproducibility values.

### 2.6. Data Analysis

Principal Component Analysis (PCA) was performed on the obtained concentration dataset (267 × 9), composed of the samples reported in Table 1, including the three replicate extracts (Section 2.2) for each sample. The samples varied according to three factors: year of cultivation (2018–2020), cut (1st, 2nd, 3rd and 4th) and basil variety (12 varieties).

Data were autoscaled to allow each of the nine molecules to contribute to the model independently of being a major or minor component.

ANOVA–Simultaneous Component Analysis (ASCA) method [24] was used to evaluate the potential significance of the effect of the three above-mentioned factors and their interactions. ASCA performs a classical ANOVA, partitioning the variability of the data into the contribution of each factor and interaction:

(1) $$X_{c}=X-1m^{T}=X_{1}+X_{2}+X_{3}+X_{1x2}+X_{1x3}+X_{2x3}+X_{1x2x3}+X_{res}$$

where *X* is the scaled data matrix, *m^T^* is the mean profile of the samples, *X* (1, 2 and 3) are the matrices related to the main effects, and *X* (1 × 2, 1 × 3, 2 × 3 and 1 × 2 × 3) are the matrices linked to the interaction effects. The rows of these matrices are highly structured, e.g., all rows related to one level (as an example 2019, for the factor year) are equal in *X*_1_ and analogously all rows of *X*_2_ and *X*_3_ are equal for each cut and type of variety. Interaction matrices also have equal rows for the same level of interaction. *X_res_* hold the residuals.

Then, each matrix was analysed by a distinct PCA model and Equation (1) can be reformulated as:

(2) $$X_{c}=T_{1}P_{1}+T_{2}P_{2}+T_{3}P_{3}+T_{1x2}P_{1x2}+\cdots +X_{res}$$

where *T* holds the scores and *P* the loadings of each PCA model, the maximum number of PCs for each model is equal to the number of levels minus one.

In order to better inspect the ASCA results, i.e., to highlight how the samples are dispersed around the mean of each effect level, it is useful to project the single samples on the ASCA scores plot. This can be achieved by adding the residuals to the estimated *x_i_* values and then calculating the single sample scores, i.e., for each factor or interaction (*f*), a computation of the score vector *t_i_*_+*res*_(*f*) has been carried out through the following equation:

(3) $$t_{i+res}\left(f\right)=\left(X_{i}\left(f\right)+X_{res}\right)p_{res}\left(f\right)$$

where *X_i_*(*f*) is the effect matrix for a specific factor or interaction and and *X_res_* is the residuals matrix, whereas *p_res_*(*f*) represents the loadings vector of the SCA model for the effect of that factor or interaction.

Since ASCA requires a balanced design of experiments to work properly, just 12 different conditions were selected from the whole dataset, leading to a total of 36 experiments as shown in Table 4. In fact, at the beginning of experimentation, a balanced design was not undertaken, also due to the limited availability of varieties which could be cultivated by the single producers; thus, it was not possible to study all levels for each of the experimental factors.

Therefore, a balanced a posteriori design was built considering two levels for the factors “year of cultivation” (2019 and 2020) and “cut” (second and fourth) and three levels for the factor “variety” (Italiano Classico, Variety 5 and Variety 9). The significance of the effect of each design factor or interaction was assessed by means of permutation tests with 1000 randomizations [39,40].

#### Software

Data analysis was performed using routines and toolboxes developed in the Matlab 2020b environment (the Mathworks Inc., Natick, MA, USA). Principal component analysis has been carried out by PLS-Toolbox v. 8.9 (Eigenvector Inc., Manson, WA, USA). ASCA has been carried out by using routines developed and kindly made available by Dr. F. Marini, University of Roma La Sapienza (Italy).

## 3. Results and Discussion

### 3.1. Aroma Analysis

**Figure 2:**
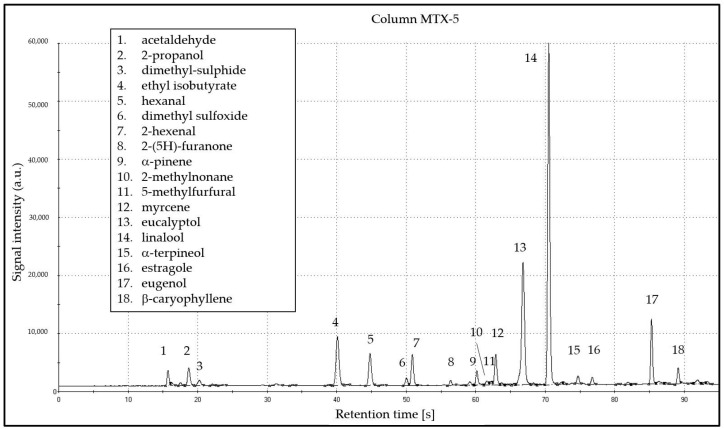
An example chromatogram obtained by elution on column MXT-5 of Heracles II. Peak 4 is the internal standard.

The pattern of volatile compounds of basil highlighted by the fast-CG analysis comprises eighteen molecules that were tentatively identified by using the Kovats relative retention indexes. The Heracles software compares the retention indexes of the two columns of different polarities to improve the tentative identification. In Figure 2, the identified molecules are shown. Among them, there are the nine ones that were identified as relevant in terms of persistent perceived odour, thus indicating that the fast-CG technique is suitable to characterise basil aroma.

The identification of these nine molecules was confirmed by comparison with the elution time of injected standards and, once quantified, their concentrations were consistent with a typical “eucalypt” basil volatile pattern [6,8] with the prevalence of linalool, followed by eucalyptol (1,8-cineole) and then by eugenol. Other molecules are typical of essential oils of basil such as hexanal, α-pinene, myrcene and caryophyllene [41].

As previously reported [7], the flavour profile is strictly related to the presence or the prevalence of key odorant molecules, with a consequent impact on the final perceived bouquet. Four main basil chemotypes have been described by Lawernce et al. [42] depending on the prevalence of odorant molecules: estragole rich, linalool rich, methyl-eugenol rich and methyl cinnamate rich. Varieties used in the present study held predominantly in the linalool rich chemotype, but with some diversity. Variety 8, for example, was characterized for its lower level of linalool compared to other varieties, whereas on the contrary, variety 9 had the higher value. In a similar way, estragole was relatively more present in varieties 8 and 9 with respect to other varieties.

### 3.2. Multivariate Exploratory Analysis

**Figure 3:**
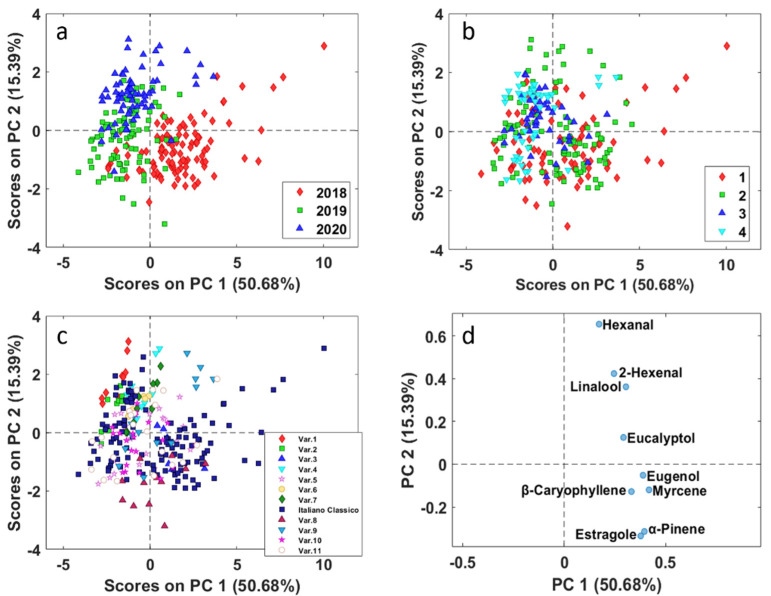
PCA of all basil samples (Table 1). PC1 vs. PC2 scores (**a**–**c**) and loadings (**d**) plots. Basil samples are coloured according to: (**a**) year; (**b**) cut; (**c**) variety.

PCA analysis was applied to the autoscaled data matrix composed by the nine volatile molecules obtained for the 267 samples characterized by different varieties, cuts and harvested years. Autoscaling was selected as the most appropriate data preprocessing method as the different volatile compounds had different variances due to their different concentration ranges. In this first exploratory analysis, two principal components seemed appropriate considering their explained variance (Figure 3).

In Figure 3, the PC1 vs. PC2 scores plot is reported and the different basil samples are represented with different symbols and colour according to year (Figure 3a), cut (Figure 3b) and basil variety (Figure 3c).

From the PCA results some information could be obtained. In particular, Figure 3a shows that slight differences could be observed among the three harvesting years, more in 2018 than in 2019 and 2020. The main contribution to this separation seems to be given by a higher concentration of almost all the investigated volatile molecules, since they lie on the same side of the respective loadings plot, all at positive values (Figure 3d). This difference is within the expected yearly variability, due to the different weather conditions. As an example, the year 2018 was probably characterized by less rainfall than in the years 2019 and 2020. As far as different basil cuts are concerned, Figure 3b points out that well defined clusters are not observable with respect to different basil cuts. Cut number 4, located on the left of the scores plot, is more homogeneous, at first it seems that the average level of all the flavour molecules is lower than in the other cuts; however, this information overlaps with that of the year.

In Figure 3c, the different varieties are rather overlapped, and it is evident a “spread” of Italiano Classico basil variety samples, which are uniformly distributed along the variability range of the scores space. Notwithstanding, PC2 highlights the difference of basil variety 8, which has the most negative scores on PC2 and thus presents a higher value of estragole and alfa-pinene (negative loadings values on PC2). A few samples harvested in 2020 of varieties 1, 4 and 9, and of Italiano Classico harvested in 2018, show high positive scores value on PC2, corresponding to higher amount of hexanal (most positive loadings value on PC2), whose odour is described as “green grass”, and could give, depending on its concentration, an unwanted “hay” note.

Finally, it can be observed that varieties 1, 2, 4, 6 and 7, which were cultivated only in 2020, are mostly located in the first quadrant (negative PC1 and positive PC2 score values) this indicates a lower amount of estragole, alfa-pinene, myrcene, b-caryophyllene, and eugenol, which fall in the opposite quadrant in the loadings space (positive PC1 and negative PC2 loading values) and thus less fruity/floral and spicy odours.

In general, the interpretation of the overall PCA results is hampered due to the combined effects of all the investigated factors.

For these reasons, after this preliminary investigation, ASCA methodology was applied on the balanced reduced dataset (Table 4) with the aim to assess if the considered experimental factors and their interactions could have a significant effect on basil’s aromatic profile. As a first step, ASCA performs a partition of the data variability into the contribution of each factor and interaction. In this case, the variation of the original data matrix was partitioned in eight different submatrices: three describing the main effect of each experimental factor—year, cuts and variety; three corresponding to the effect of each second order interaction (any possible combination of levels for each couple of factors); one accounting for the three-way interaction effect (not considered in this study), and one holding the residuals. The significance of the factors or interactions’ effects was assessed by means of a permutation test, which compares the experimental sum of squares for each effect matrix with its corresponding distribution under the null hypothesis. Results of the test are shown in Table 5, where the explained variance and probability *p*-value are reported for each factor and their second order interaction. All the considered factors and interactions were statistically significant (*p* < 0.05), even though the effect of the factors “variety” and “year” presented a higher explained variance than other effects. On the other hand, the effect of factor “cut” explained just 3% of the total variance, suggesting a lower influence on basil’s aromatic profile compared with the other two main factors. This can also be seen in the fact that the second order interactions in which factor “cut” is involved explain less than the 4% of the total variance, whereas the interaction “year × variety” explains almost 12%.

**Figure 4:**
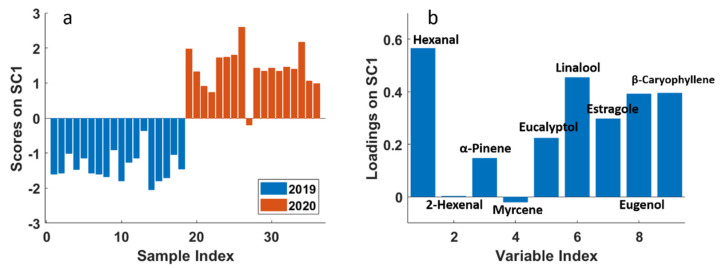
SCA of the effect matrix “year”. (**a**) Scores plot (SC1) with projected residuals; (**b**) variable loadings (SC1).

After the assessment of the significance of each factor and interaction, a component analysis (SCA) was performed on each effect matrix separately in order to interpret the observed variation. In Figure 4a, the scores plot of the effect for factor “year”, with projected residuals, is shown. This plot was obtained according to Equation (3). Since the year effect matrix contains just two rows, one for each considered year, the SCA model is described by only one component (SC1), which explains 100% of the variance.

From the scores plot, it was possible to confirm the significant difference between the two levels of the factor “year”: all samples collected in 2019 have negative scores, whereas almost all the samples collected in 2020 have positive scores, highlighting the high difference between the two levels of this factor. To explain this difference, in Figure 4b the corresponding loadings plot is reported, where it can be observed that the year 2020 samples present higher contents of almost all the molecules investigated in the study, except for 2-hexenal and myrcene, which do not contribute to explain the difference between the two years.

**Figure 5:**
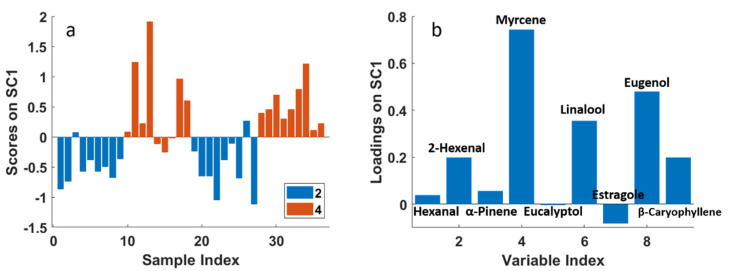
SCA of the effect matrix “cut”. (**a**) Scores plot (SC1); (**b**) variable loadings (SC1).

Figure 5a,b shows the scores and loadings plots for the effect of factor “cut”, respectively. They are represented in the same way as for the factor “year”. In this case, the scores plot confirms that there is a significant difference between the second and fourth cuts, even if it is not as marked as for the other main factors. In particular, scores of samples from 10 to 18 (4th cut, year 2019) present both positive and negative values in an irregular pattern. From the loadings plot, it is possible to observe that samples collected at the fourth cut present mainly a higher content of myrcene, eugenol and linalool, with respect to the second cut samples. β-caryophyllene and 2-hexenal contribute in the same direction but to a lesser extent. A slightly lower content of estragole characterizes the second cut. In general, for the factor “cut”, not all the samples characterized by the same conditions behave similarly, as the effect of “cut” is of the same entity of its interactions with year and variety, as highlighted in Table 4. However, the general trend suggests that the influence of this factor on basil’s aromatic profile cannot be neglected.

**Figure 6:**
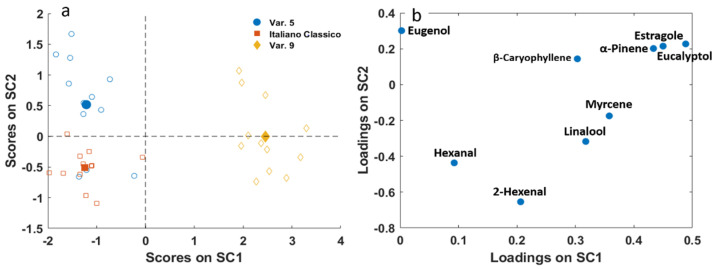
SCA of the effect matrix “variety”. (**a**) SC1 vs. SC2 scores plot with projected residuals (empty symbols); (**b**) variable loadings (SC1 vs. SC2).

Results of SCA for the factor “variety” are represented in Figure 6. In this case, since the factor “variety” was varied at three levels, two components (SCs) were necessary to describe its effect. The first SC clearly describes the difference between Var. 9 with respect to Var. 5 and Italiano Classico varieties. Var. 9 presented a higher content of almost all the molecules considered in this study, especially eucalyptol, estragole, and α-pinene, which gave a balsamic connotation to the odour (Table 2). On the other hand, the second SC shows the difference between Var. 5 and Italiano Classico varieties, less marked than the difference described by SC1. In this case, the compounds mainly responsible for this difference are hexanal and 2-hexenal, which are in greater quantity in the Italiano Classico variety, whereas Var. 5 is characterized by slightly higher quantities of eugenol, β-caryophyllene, α-pinene, estragole and eucalyptol.

**Figure 7:**
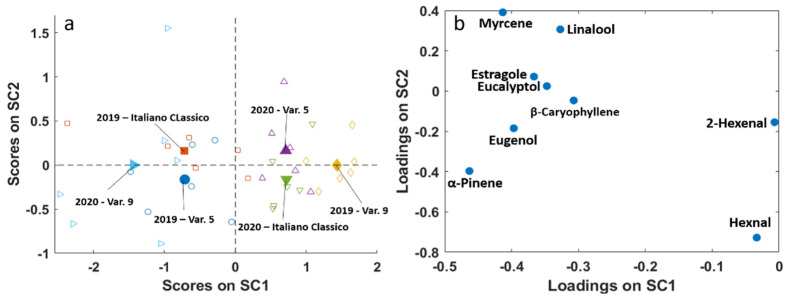
SCA of the effect matrix interaction “year x variety”. (**a**) SC1 vs. SC2 scores plot with projected residuals (empty symbols); (**b**) variable loadings (SC1 vs. SC2).

To deeply investigate the effect of considered factors on basil’s aromatic profile, their second order interactions were also examined. Figure 7 shows the effect of the interaction between the factors “year” and “variety”. It is possible to observe how Var. 9 is extremely different from the other two varieties, as it shows the opposite behaviour in SC1, i.e., Var. 9 samples collected in 2020 (negative SC1 values) have a higher content of almost all the considered molecules (negative SC1 loadings, except for 2-hexenal and hexanal close to zero) with respect to samples of the same variety collected in 2019. At variance, the other two varieties are richer in flavours in 2019 than in 2020. Italiano Classico and Var. 5 show the opposite behaviour with respect to year in SC2: the first is richer in flower/fruity aroma (higher myrcene and linalool) and lower in α-pinene and hexanal in 2019 with respect to 2020, and the opposite holds for Var. 5. Thus, it is worth noting how the variation of the factor “year” changes the chemical composition of samples of the same variety.

**Figure 8:**
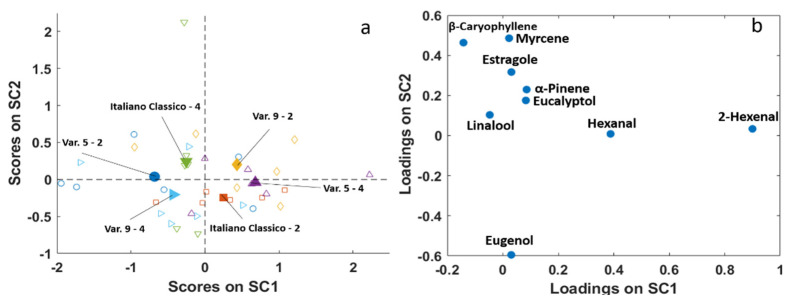
SCA of the effect matrix interaction “cut x variety”. (**a**) SC1 vs. SC2 scores plot with projected residuals (empty symbols); (**b**) variable loadings (SC1 vs. SC2).

The same pattern can be observed in Figure 8, which describes the effect of the interaction between the factors “cut” and “variety”. In this case, the variation of factor “cut” is the one that strongly changes the chemical composition of samples characterized by the same variety, even if it does it to a lesser extent than the factor “year”. High SC1 values correspond to a high 2-hexenal content, whereas low SC2 values are linked to high eugenol values.

Considering the projected residuals, the differences are appreciable mainly in SC1, where Italiano Classico and Var. 9 show the same behaviour, being richer in floral/fruity flavours in cut 4 with respect to 2, while the opposite holds for Var. 5.

## 4. Conclusions

The results obtained support the use of a fast-GC based electronic nose for rapid assessment of basil aroma; in fact, the main molecules perceived as persistent by olfactometry (GC/O) are identifiable and quantifiable. In agreement with previous literature, it has been observed that the aroma composition is not only a distinctive trait of variety, but the content of each specific molecule varies with agronomic year and cut period. On the one hand, this renders more problematic the choice of a specific variety to be cultivated to achieve a desired flavor profile; on the other hand, it may help focus on the varieties showing more stability with respect to the agronomic variability. In terms of percentage of variance, the cut affects the aroma less with respect to year and variety. The effect of year seems to be a bulk effect affecting the content more than the type of molecules found in the aroma.

## Figures and Tables

**Table 1:** Samples analysed in the three years of experiment with the indication of the samples undertaken for each cut.

Crop Year	Basil Variety	Cut in Bold (No. of Samples)
2018	italiano classico variety 3 variety 5 variety 8 variety 10 variety 11	**1st** (11), **2nd** (12), **3rd** (3) **1st** (1), **2nd** (1) **1st** (1), **2nd** (1) **1st** (1), **2nd** (1) **1st** (1), **2nd** (1) **1st** (1), **2nd** (1)
2019	italiano classico variety 5 variety 8 variety 9 variety 10 variety 11	**1st** (4), **2nd** (2), **3rd** (2), **4th** (2) **1st** (2), **2nd** (1), **3rd** (1), **4th** (1) **1st** (2) **2nd** (1), **3rd** (1), **4th** (1) **1st** (2), **2nd** (1), **3rd** (1), **4th** (1) **1st** (2), **2nd** (1), **3rd** (1), **4th** (1)
2020	italiano classico variety 1 variety 2 variety 4 variety 5 variety 6 variety 7 variety 9	**2nd** (2), **3rd** (1), **4th** (2) **2nd** (1), **3rd** (1), **4th** (1) **2nd** (1), **3rd** (1), **4th** (1) **2nd** (1), **3rd** (1), **4th** (1) **2nd** (1), **4th** (1) **3rd** (1), **4th** (1) **2nd** (1), **3rd** (1), **4th** (1) **2nd** (1), **4th** (1)

**Table 2:** Persistent molecules found in basil aroma, selected by GC/O, with CAS Number and the descriptions assigned by the CC-O panelists.

Molecules	CAS Number	Aroma Description
hexanal	66-25-1	green grass, rancid
2-hexenal	63449-41-2	spices/herbal
a-pinene	80-56-8	herbal, woody
b-myrcene	123-35-3	flower, cytrus
eucalyptol	470-82-6	balsamic, eucalyptus, menthol
linalool	78-70-6	flower, cytrus, vinegar
estragole	140-67-0	anis, liquorice, fennel
eugenol	97-53-0	cloves, spices
b-caryophyllene	87-44-5	spices

**Table 3:** Coefficient of determination (R^2^), slope of the calibration curves, and limit of detection for the investigated compounds.

Compounds	R^2^	Slope ± SD	LOD (µg kg^−1^)
hexanal	0.9997	0.96 ± 0.01	47
2-hexenal	0.9998	0.79 ± 0.01	23
a-pinene	0.9998	1.73 ± 0.01	28
b-myrcene	0.9999	1.61 ± 0.01	11
eucalyptol	0.9999	1.88 ± 0.01	22
linalool	0.9995	0.394 ± 0.004	60
estragole	0.9994	1.33 ± 0.02	52
eugenol	0.9999	0.453 ± 0.002	32
b-caryophyllene	0.9968	1.22 ± 0.03	22

**Table 4:** Design of experiments structure for ASCA.

Year	Cut	Variety
2019	2	Variety 5
2019	2	Italiano Classico
2019	2	Variety 9
2019	4	Variety 5
2019	4	Italiano Classico
2019	4	Variety 9
2020	2	Variety 5
2020	2	Italiano Classico
2020	2	Variety 9
2020	4	Variety 5
2020	4	Italiano Classico
2020	4	Variety 9

**Table 5:** Explained variance and probability values for main factors and their second order interactions.

Factor	Expl. Var. %	*p*
Variety	36.41	<0.001
Year	22.31	<0.001
Year × Variety	11.95	<0.001
Year × Cut	3.74	<0.001
Cut × Variety	3.1	0.003
Cut	3	<0.001

## Data Availability

The data are available on request from the authors.

## Sample Availability

Not available.
